# Short- and long-term cause-specific survival of patients with inflammatory breast cancer

**DOI:** 10.1186/1471-2407-5-137

**Published:** 2005-10-22

**Authors:** Patricia Tai, Edward Yu, Ross Shiels, Juan Pacella, Kurian Jones, Evgeny Sadikov, Shazia Mahmood

**Affiliations:** 1 University of Saskatchewan, Faculty of Medicine, Saskatoon; Department of Radiation Oncology, Regina, Canada; 2 Division of Radiation Oncology, Department of Oncology, University of Western Ontario, London, Ontario, Canada

## Abstract

**Background:**

Inflammatory breast cancer (IBC) had been perceived to have a poor prognosis. Oncologists were not enthusiastic in the past to give aggressive treatment. Single institution studies tend to have small patient numbers and limited years of follow-up. Most studies do not report 10-, 15- or 20-year results.

**Methods:**

Data was obtained from the population-based database of the Surveillance, Epidemiology, and End Results program of the National Cancer Institute from 1975–1995 using SEER*Stat5.0 software. This period of 21 years was divided into 7 periods of 3 years each. The years were chosen so that there was adequate follow-up information to 2000. ICD-O-2 histology 8530/3 was used to define IBC. The lognormal model was used for statistical analysis.

**Results:**

A total of 1684 patients were analyzed, of which 84% were white, 11% were African Americans, and 5% belonged to other races. Age distribution was < 30 years in 1%, 30–40 in 11%, 40–50 in 22%, 50–60 in 24%, 60–70 in 21%, and > 70 in 21%. The lognormal model was validated for 1975–77 and for 1978–80, since the 10-, 15- and 20-year cause-specific survival (CSS) rates, could be calculated using the Kaplan-Meier method with data available in 2000. The data were then used to estimate the 10-, 15- and 20-year CSS rates for the more recent years, and to study the trend of improvement in survival. There were increasing incidences of IBC: 134 patients in the 1975–77 period to 416 patients in the 1993–95 period. The corresponding 20-year CSS increased from 9% to 20% respectively with standard errors of less than 4%.

**Conclusion:**

The improvement of survival during the study period may be due to introduction of more aggressive treatments. However, there seem to be no further increase of long-term CSS, which should encourage oncologists to find even more effective treatments. Because of small numbers of patients, randomized studies will be difficult to conduct. The SEER population-based database will yield the best possible estimate of the trend in improvement of survival for patients with IBC.

## Background

Inflammatory breast cancer (IBC) occurs rarely [1]. Signs and symptoms of this condition include the presence of erythema, edema or peau d'orange appearance of the skin, and other clinical signs of disease. Diagnosis is made by skin biopsy. The definition of IBC varies in the literature and leads to some disparities. In this study, the pathological definition is used.

It is known that IBC have a poor prognosis. Oncologists were not enthusiastic to administer aggressive treatment in the past. Nowadays, treatment for this aggressive form of breast cancer is multi-modal, and includes chemotherapy, surgery, radiation therapy, and hormonal therapy [2]. The optimal sequence of the different modalities is still a subject of research [3]. Development of novel therapeutic agents continues and is based on an expanding understanding of the biology of tumor development and progression. Advances in treatment continue to improve the prognosis for this disease [4]. With a few notable exceptions, many publications on IBC do not have long periods of follow-up [5,6]. These single-institution studies are from academic centers. To our knowledge, long-term results of cases treated in the community are not available. This study examines the changes in the prognosis of IBC over the years with the Surveillance, Epidemiology, and End Results (SEER) database [7].

There is a parametric lognormal model, proposed by Boag [8-10] that has been validated retrospectively in the literature, and can be used prospectively for predicting long-term survival rates several years earlier than would otherwise be possible using the Kaplan-Meier method of calculation [11].

Boag's lognormal model for long-term cancer survival rates has been available for use for some 50 years. When the lognormal model was first proposed in the 1940s, it was difficult to implement because of a lack of computing power, and lack of good quality long-term follow-up data from cancer registries. Since 1970s the model has been used by authors on breast cancer, cervix uteri cancer, head and neck cancer, intraocular melanoma, choroidal-ciliary body melanoma, and small cell lung cancer [12-17]. Currently, although available computing power is adequate, good quality follow-up data on a sufficient number of patients are seldom available, and so can limit the application of Boag's model. Studies from single institutions tend to have small number of patients and limited years of follow-up for IBC. Use of a large data registry such as the SEER database with good long-term follow-up data can overcome these potential limitations.

## Methods

From the population database of the Surveillance, Epidemiology, and End Results program of the National Cancer Institute from 1975–1995, data were extracted using SEER*Stat5.0 software from the 9 registries: San Francisco-Oakland, Connecticut, Metropolitan Detroit, Hawaii, Iowa, New Mexico, Seattle (Puget Sound), Utah, and Metropolitan Atlanta. This period of 21 years was divided into 7 periods of 3 years each. The years of diagnosis were 1975–77, 1978–80, 1981–83, 1984–86, 1987–89, 1990–92, and 1993–95. These years were chosen so as to provide adequate follow-up information to 2000. ICD-O-2 histology 8530/3 was used to define IBC. The data used in the study were survival time, vital status, and cause of death.

The cause-specific survival (CSS) was defined as the interval from the date of diagnosis to the date of death from breast cancer or to the last follow-up date for censoring purposes, if the patient was alive and was still being followed at the time of data cut-off.

The lognormal model was used for statistical analysis. Using short-term follow-up data, the lognormal model can predict long-term survival rates comparable in accuracy with those calculated by the Kaplan-Meier method using long-term follow-up [18]. The assumption of the lognormal model is that the survival times of the patients died of a specific cancer follows a lognormal distribution. What lognormal distribution means is that it becomes a normal distribution when the variables are converted by taking logarithmic transformation. The lognormal model has three parameters: the standard deviation S, the mean M and the proportion cured C. The proportion cured is defined as the portion of all the patients treated remaining alive and symptom free for a long period, some of those who died of intercurrent diseases are presumably cured of the cancer. This lognormal model used a maximum likelihood method to estimate long-term CSS (e.g., 10-year, 15-year and 20-year survival rates) from only short-term follow-up data. The CSS rates at time τ is calculated as [C+ (1-C)·Q]·100%, where C is the proportion cured of patients and Q is the integral of the lognormal distribution between the limits of time τ and infinity.

The long-term survival rates were predicted by Boag's method using a computer program run by Microsoft Excel. In this parametric lognormal model, the standard deviation S was fixed; only the two remaining parameters, mean M and proportion cured C, were kept floating when using the maximum likelihood method. A range (0.35–0.55) of S with step 0.01 was tested. The value of S was chosen for the best fit to the first five years known survival curve obtained by the Kaplan-Meier method, and also multiple iterations converged to a stable solution for M and C. The parameters obtained are shown in Table 1.

A 3-year period of diagnosis was selected and patients were followed as a cohort for an additional 3 years. For example, for cases diagnosed during the 3-year period, 1975–1977, prediction of the long-term survival rates was made using follow-up data to December 31, 1980 (i.e., 3 years after 1977). The predicted long-term survival rates for patients diagnosed during 1975–1977, and 1978–1980 were compared to the Kaplan-Meier estimates.

Confidence intervals are calculated by +/- 1.96 (standard error), assuming that the errors are normally distributed.

## Results

A total of 1684 patients were extracted from the SEER database: 84% were white, 11% were African-Americans, and 5% belonged to other races. Age distribution was < 30 years in 1%, 30–40 in 11%, 40–50 in 22%, 50–60 in 24%, 60–70 in 21%, and > 70 in 21%. Table 2 shows the patient characteristics of the 7 periods in the study.

The proportions cured as shown in Table 1 for the different periods are almost linearly increasing (correlation coefficient of determination, R^2 ^= 0.93) across the years. The number of breast cancer deaths and the number of total deaths for the 7 periods in the study are shown in Table 3 at different time of follow-up.

**Figure 1:**
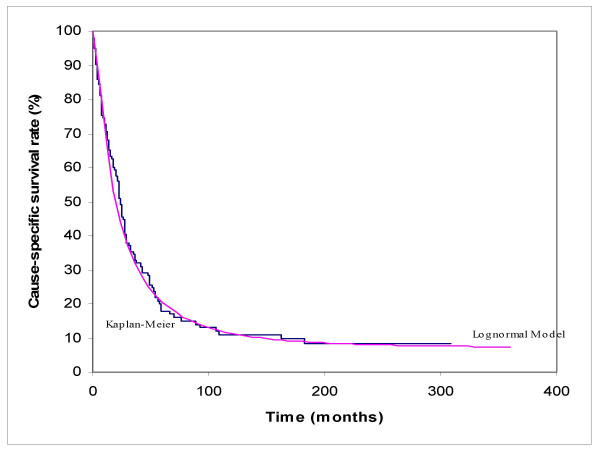
Fitting of the lognormal model estimation to the Kaplan-Meier curve for 1975–1977.

**Figure 2:**
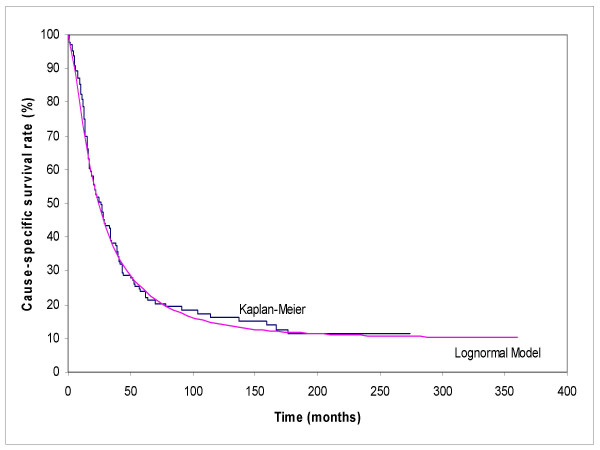
Fitting of the lognormal model estimation to the Kaplan-Meier curve for 1978–1980.

**Figure 3:**
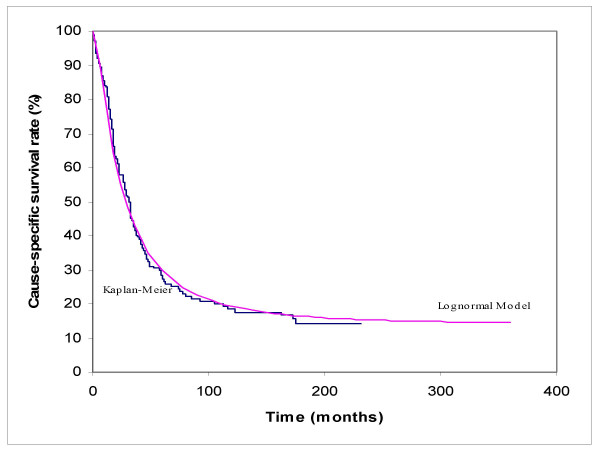
Fitting of the lognormal model estimation to the Kaplan-Meier curve for 1981–1983.

**Figure 4:**
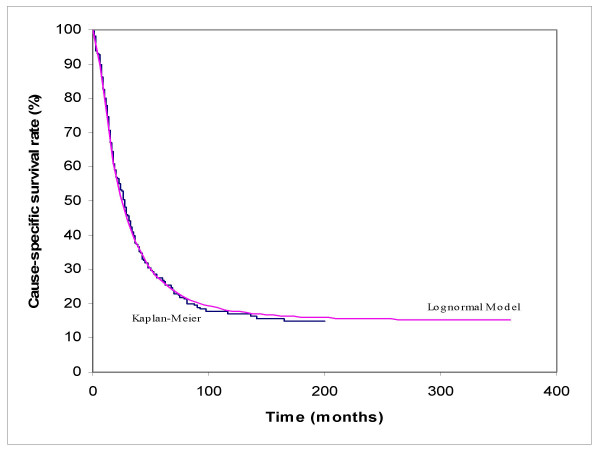
Fitting of the lognormal model estimation to the Kaplan-Meier curve for 1984–1986.

**Figure 5:**
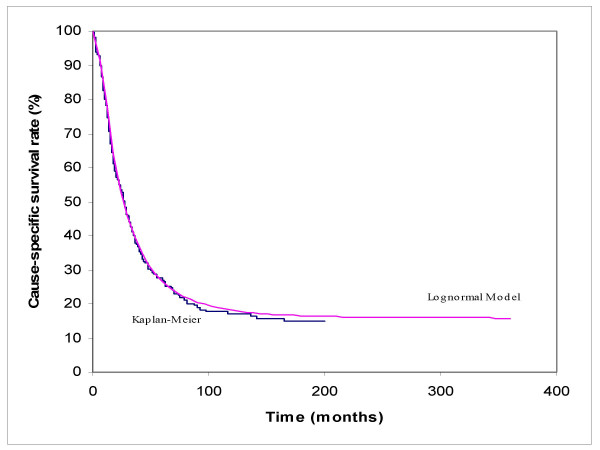
Fitting of the lognormal model estimation to the Kaplan-Meier curve for 1987–1989.

**Figure 6:**
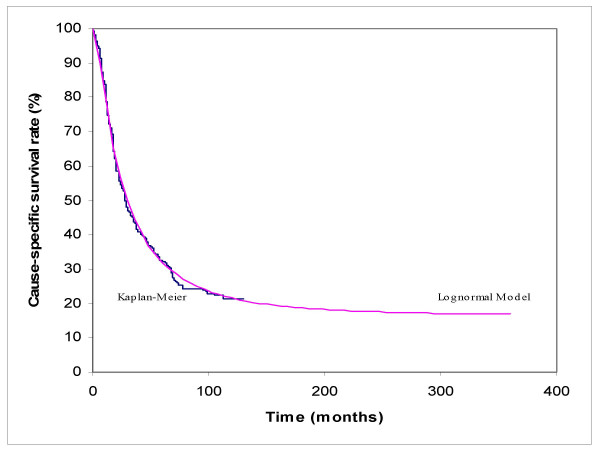
Fitting of the lognormal model estimation to the Kaplan-Meier curve for 1990–1992.

**Figure 7:**
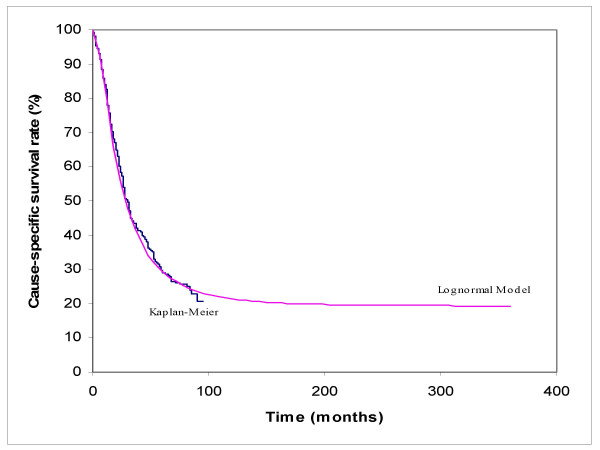
Fitting of the lognormal model estimation to the Kaplan-Meier curve for 1993–1995.

The 5-, 10-, 15-, and 20- year CSS by period of diagnosis, estimates by the lognormal model and the non-parametric Kaplan-Meier method if applicable are shown in Table 4. The standard errors were less than 4%. For patients diagnosed in 1975–77, the 5-year CSS was 18% and 20-year CSS was 9%. In the modern era, 1993–95, the 5-year CSS increased to 29% and 20-year CSS was estimated to be 20%. Table 5 shows the short-term CSS comparison obtained by the Kaplan-Meier method and the lognormal model. Figures 1, 2, 3, 4, 5, 6, 7 show both lognormal model estimations and Kaplan-Meier curves for the different periods.

## Discussion

### IBC and its outcome after treatment

IBC is a distinct entity different from the usual locally advanced breast cancer. Chang *et al*. [19] studied the incidence of IBC in the SEER database. IBC patients were significantly younger at diagnosis than non-IBC patients. Among both IBC and non-IBC patients, African Americans were younger than whites. Overall survivals (OS) were significantly worse for IBC patients than for non-IBC patients and for African Americans than for whites. Among whites, the 3-year survival improved more for IBC patients than for non-IBC patients between 1975–1979 and 1988–1992, increasing from 32% to 42% for IBC patients (P = 0.0001) and from 80% to 85% for non-IBC patients (P = 0.0001).

Low *et al*. [20] compared IBC versus non-IBC among patients in the National Cancer Institute. The 46 IBC patients had a median overall survival of 3.8 years and event free survival of 2.3 years, compared with 12.2 and 9.0 years, respectively, in stage IIIA breast cancer patients. Fifteen-year OS survival was 20% for IBC versus 50% for stage IIIA patients and 23% for stage IIIB non-IBC patients.

Table 6[3,6,21-28] summarizes the key results in research for IBC. Chang *et al*. [22] evaluated the effects of obesity and menopausal status on survival in a cohort of 177 female IBC patients diagnosed between 1974 and 1993. They found that factors associated with larger body size at diagnosis may contribute to shorter survival among postmenopausal IBC women but not among pre-menopausal IBC women. The latter were found to have poor survival regardless of body size.

In the table of patient characteristics (Table 2) of the different periods, the age distributions are similar for the different periods. However, the earlier 3 periods have similar stage distribution, and the later 4 periods have another similar stage distribution, with more distant stage patients compared with the earlier 3 periods. Despite an increasing proportion of distant stage, survival is increasing. This likely reflects the vigilance of staging investigations in recent time periods.

The present study shows a gradual increase in CSS rate over time, for both the Kaplan-Meier method and the lognormal model. The estimations of the long-term CSS rates by the lognormal model for 1975–77 and 1978–80 were validated within one standard error of those rates calculated by using Kaplan-Meier method. The above results show what is achievable in different institutions over a wide area of United States. Published single institution studies from major or tertiary referral centers do not reflect the true picture of care in the community. The long-term CSS calculated by the lognormal model for the cohort diagnosed in the years 1993–1995 is stable at 20%. There is little further drop of CSS beyond 10 years. The improvement in survival has been achieved by years of research, as noted below.

Perez *et al*. [23] analyzed 179 patients with histologically confirmed inflammatory carcinoma of the breast. Minimum follow-up was 2 years (maximum, 12 years; median, 4 years in the surviving patients). Clearly better loco-regional tumor control, i.e. in the breast and regional nodal drainage area, was observed in patients who underwent a surgical procedure: 79% with three modalities, 76% with irradiation and surgery, and only 30% with irradiation alone or in combination with chemotherapy. The addition of mastectomy to irradiation significantly improved loco-regional tumor control, disease free survival (DFS), and CSS. The combination of chemotherapy, surgery, and irradiation had a significant impact on loco-regional tumor control and incidence of distant metastases compared with surgery plus irradiation, and a lesser impact on DFS and CSS.

The literature indicates that chemotherapy does not negate the importance of radiation in optimizing loco-regional control in patients with high-risk breast cancer. The results of recent randomized trials studying postmastectomy radiation show that improved loco-regional control improves OS. Thus many authors believe that all breast cancer patients who have high-risk primary breast cancer and who are treated with chemotherapy should receive radiation as a component of their treatment [26]. Liao *et al*. [27] studied 115 patients with nonmetastatic IBC and tested the use of twice a day (b.i.d.) radiotherapy treatment with total dose of 60 Gy and 66 Gy at different time periods. Accelerated, b.i.d. fractionation at 1.5 Gy per fraction to a dose of 45 Gy plus 15 Gy chest wall boost was used for most patients from 1982 to 1985. From 1986–1993, 51 Gy in 34 fractions was delivered over 17 treatment days, followed by a 15 Gy chest wall boost in 10 fractions over a period of 5 treatment days. Chemotherapy regimens used did not change significantly during the period of that study. Long-term complications of radiation, such as arm edema of more than 3 cm (in 7 patients), rib fracture (in 10 patients), severe chest wall fibrosis (in 4 patients), and symptomatic pneumonitis (in 5 patients), were comparable in the two groups of 60 Gy versus 66 Gy, indicating that the dose escalation did not result in increased morbidity. Significant differences in the rates of loco-regional control (P = 0.03) and OS (P = 0.03), and a trend towards better DFS (P = 0.06) were observed among those recently treated patients who received higher doses of irradiation. For the entire patient group who received radiotherapy either once or twice daily, the 5- and 10-year local control rates were 73.2% and 67.1%, respectively. The 5- and 10-year DFS were 32.0% and 28.8%, respectively, and the overall survival rates for the entire group were 40.5% and 31.3%, respectively.

In France, a study on the impact of intensity of chemotherapy was performed [28] on 74 women with nonmetastatic IBC consecutively treated between 1976 and 2000. Patients received primary anthracycline-based chemotherapy either at conventional doses (n = 20) or at high does with hematopoietic stem cell support (HSCS) (n = 54). In multivariate analysis, the strongest independent prognostic factor was the delivery of high-dose chemotherapy (HDC). The 5-year DFS and OS of patients were respectively 28% and 50% with HDC and 15% and 18% with conventional chemotherapy. These results suggest that HDC with HSCS may have a role in the treatment of IBC.

Recent study on the use of trastuzumab and paclitaxel may well lead to further research on the use of different combinations of chemotherapy and biological response modifiers [29] for the treatment of IBC. After completion of chemotherapy, for patients whose tumors showed receptor-positive tumors; additional tamoxifen therapy (if post-menopausal) or gonadotropin-releasing hormone (GnRH)-analogues (if pre-menopausal) were given. However, it is still uncertain, whether better prognosis can be achieved by treatment with GnRH-analogues [30].

Ueno *et al*. [6] found from the long-term follow-up data on patients treated with a combined-modality (chemotherapy, then mastectomy, then chemotherapy and radiotherapy) approach, a significant fraction of patients (estimated to be 28%) remained free of disease beyond 15 years. There were virtually no recurrences after 10 years. Estimated DFS at 5 years was 32%, at 10 years was 28%, and at 15 years was 28%. Estimated OS at 5 years was 40%, at 10 years was 33%, and at 15 years was 29%. By contrast, single-modality treatment (radiotherapy or surgery alone) gave a DFS of less than 5% beyond 15 years. Thus, combined-modality treatment is recommended as the standard of care for IBC.

### Possible explanations to the improved survival

IBC is a distinct clinicopathologic entity separate from noninflammatory locally advanced breast carcinoma [31,32]. Improvements in population-based survival represent the extent to which therapies with demonstrated efficacy are translated to the real population. Thus, they represent the effect of dissemination of new therapies and effectiveness. In the early 1970s, the commonly used regimen was FAC (5-fluorouracil, adriamycin, cyclophosphamide) before and after radiotherapy. In the late 1970s, FAC was given before and after mastectomy [6]. Taxanes become increasingly used in America since 2001 [33].

There are several other possible explanations to the improved survival other than treatment changes: change of the definition and classification of IBC, proportion of cause specific deaths not based on autopsy, change of patient population (age distribution, stages [IIIB and IV]). Obesity is a poor prognostic factor and so the improvement of IBC survival is not related to increasing obesity noted in the American population. Better treatment and the above factors all account for the improved survival.

## Conclusion

The improvement of survival during the study period may be due to introduction of more aggressive treatments. However, there seem to be no further increase of long-term CSS, which should encourage oncologists to find even more effective treatments. Because of small numbers of patients, randomized studies will be difficult to conduct. The SEER population-based database will yield the best possible estimate of the trend in improvement of survival for patients with IBC.

## List of abbreviations

C: Proportion cured

CSS: Cause-specific survival

DFS: Disease-free survival

IBC: Inflammatory breast cancer

M: Mean

OS: Overall survival

S: Standard deviation

SEER: Surveillance, Epidemiology, and End Results

## Competing interests

The author(s) declare that they have no competing interests.

## Authors' contributions

PT: Data analysis and writing of the manuscript.

EY, RS, JP, KJ, ES, SM: Critical appraisal of the manuscript.

All authors read and approved the final manuscript.

## Pre-publication history

The pre-publication history for this paper can be accessed here:



## Figures and Tables

**Table 1:** Parameters in the lognormal model estimations with standard errors in brackets.

Years of diagnosis	Selected S	Estimated M	Estimated C
1975–77	0.50	1.25(0.06)	0.07(0.06)
1978–80	0.48	1.32(0.06)	0.10(0.06)
1981–83	0.48	1.37(0.06)	0.13(0.06)
1984–86	0.43	1.30(0.04)	0.15(0.05)
1987–89	0.40	1.33(0.04)	0.16(0.04)
1990–92	0.47	1.36(0.04)	0.16(0.04)
1993–95	0.39	1.33(0.03)	0.19(0.03)

S = standard deviation,

M = mean,

C = proportion cured.

**Table 2:** Patient characteristics.

Years of diagnosis	1975–77	1978–80	1981–83	1984–86	1987–89	1990–92	1993–95
No. of patients	134	145	181	210	258	340	416
Race:							
White	117	124	162	184	207	276	352
Black	16	15	9	19	37	49	40
Others	1	6	8	7	13	15	24
Unknown	0	0	2	0	1	0	0
Age:							
Median	58	56	60	56	55	59	56
Range	26–97	29–91	22–89	25–91	25–101	34–94	26–97
Stage:							
Localized	6	3	6	1	1	5	5
Regional	84	100	101	41	27	20	21
Distant	35	37	72	161	218	304	385
Unstaged	9	5	2	7	12	11	5

**Table 3:** Number of deaths due to breast cancer and number of total deaths at six-month intervals for the 7 periods of diagnosis.

	1975–77		1978–80		1981–83		1984–86		1987–89		1990–92		1993–95	
	
Interval	BCD	TD	BCD	TD	BCD	TD	BCD	TD	BCD	TD	BCD	TD	BCD	TD
0–6	25	26	15	19	19	22	21	26	23	28	29	36	35	47
6–12	15	20	15	18	15	22	27	32	27	29	47	51	49	52
12–18	12	16	26	28	24	26	31	31	37	40	41	46	44	49
18–24	13	15	10	12	14	14	15	17	27	30	31	36	38	38
24–30	13	13	11	11	11	13	15	16	14	15	23	26	33	34
30–36	6	6	7	8	16	16	15	16	18	20	11	15	24	30
36–42	4	6	8	9	7	9	9	11	15	15	11	12	11	13
42–48	1	1	4	5	7	9	5	6	9	13	8	9	15	17
48–54	7	7	4	4	3	3	3	4	5	5	7	9	15	16
54–60	4	6	2	3	5	5	3	4	2	2	6	7	11	12
60–66	1	1	3	3	2	3	3	3	4	4	5	6	4	5
66–72	1	1	1	1	1	2	4	4	4	4	11	12	4	6
72–78	1	1	1	3	3	3	2	2	3	4	6	7	1	5
78–84	0	0	0	2	1	2	3	3	0	1	0	1	2	2
84–90	1	1	0	1	1	3	2	2	1	1	0	0	2	2
90–96	1	1	1	1	1	2	1	1	3	5	1	5	0	0
96–102	0	0	0	0	0	0	1	1	2	3	2	5		
102–108	1	1	1	1	1	1	0	1	0	0	1	1		
108–114	1	2	1	1	1	1	0	0	1	2	1	1		
114–120	0	0	0	0	1	1	1	2	0	0	0	1		
120–126	0	0	0	0	1	1	0	0	0	0	0	0		
126–132	0	0	0	0	0	0	0	0	0	1	0	0		
132–138	0	1	1	1	0	0	1	2	1	2				
138–144	0	0	0	1	0	0	1	2	0	1				
144–150	0	0	0	0	0	1	0	0	0	2				
150–156	0	0	0	1	0	1	0	1	0	1				
156–162	1	1	1	1	1	3	0	0	0	0				
162–168	0	0	1	1	0	2	1	1						
168–174	0	0	0	0	1	2	0	1						
174–180	0	0	1	1	1	1	0	2						
180–186	1	1	0	0	0	0	0	1						
186–192	0	0	0	0	0	0	0	0						
192–198	0	0	0	0	0	0	0	0						
198–204	0	0	0	1	0	1	0	0						
204–210	0	0	0	0	0	0								
210–216	0	0	0	0	0	0								
216–222	0	0	0	0	0	0								
222–228	0	0	0	0	0	0								
228–234	0	0	0	0	0	0								
234–240	0	2	0	0										
240–246	0	0	0	2										
246–252	0	0	0	1										
252–258	0	0	0	0										
258–264	0	0	0	1										
264–270	0	0	0	0										
270–276	0	0	0	0										
276–282	0	1												
282–288	0	0												
288–294	0	0												
294–300	0	0												
300–306	0	0												
306–312	0	0												

BCD = breast cancer deaths

TD = total deaths

**Table 4:** 5-, 10-, 15- and 20-year cause-specific survival (CSS) rates, in percentages, estimated by Kaplan-Meier method compared with those estimated by lognormal model [in square brackets] with 95% confidence intervals (in round brackets).

Years of diagnosis	5 yr CSS	10 yr CSS	15 yr CSS	20 yr CSS
1975–77	18(16.5–19.4), [20](18.4–21.5)	11(10.3–11.6), [11](10.3–11.6)	9(8.4–9.5), [9](8.4–9.5)	9(8.4–9.5), [8](7.5–8.4)
1978–80	23(21.1–24.8), [24](22.1–25.8)	16(15.0–16.9), [14](13.1–14.8)	11(10.3–11.6), [12](11.2–12.7)	11(10.3–11.6), [11](10.3–11.6)
1981–83	27(24.8–29.1), [30](27.6–32.2)	18(16.9–19.0), [19](17.8–20.1)	14(13.1–14.8), [15](14.1–15.8)	NA, [14](13.1–14.8)
1984–86	27(25.4–28.5), [26](24.4–27.5)	17(16.0–17.9), [18](16.9–19.0)	15(14.1–15.8), [16](15.0–16.9)	NA, [15](14.1–15.8)
1987–89	27(25.4–28.5), [27](25.4–28.5)	19(17.8–20.1), [18](16.9–19.0)	NA, [16](15.0–16.9)	NA, [16](15.0–16.9)
1990–92	32(30.1–33.8), [32](30.1–33.8)	21(19.7–22.2), [22](20.7–23.2)	NA, [19](17.8–20.1)	NA, [18](16.9–19.0)
1993–95	29(27.8–30.1), [29](27.8–30.1)	NA, [21](19.7–22.2)	NA, [20](18.8–21.1)	NA, [20](18.8–21.1)

NA = not available by Kaplan-Meier method

**Table 5:** 0.5-, 1-, 2-, 3- and 4-year cause-specific survival (CSS) rates, in percentages, estimated by Kaplan-Meier method compared with those estimated by lognormal model [in square brackets] with 95% confidence intervals (in round brackets).

Years of diagnosis	0.5 yr CSS	1 yr CSS	2 yr CSS	3 yr CSS	4 yr CSS
1975–77	81(76.2–85.7), [84](79.0–88.9)	70(64.5–75.4), [66](60.8–71.1)	49(45.1–52.8), [44](40.5–47.4)	33(30.4–35.5), [32](29.4–34.5)	28(25.8–30.1), [25](23.0–26.9)
1978–80	89(83.7–94.2), [89](83.7–94.2)	79(74.3–83.6), [73](68.7–77.2)	52(47.9–56.0), [51](47.0–54.9)	38(35.0–40.9), [38](35.0–40.9)	28(25.8–30.1), [30](27.6–32.3)
1981–83	89(85.5–92.4), [90](86.4–93.5)	81(76.2–85.7), [76](71.5–80.4)	58(53.4–62.5), [55](50.6–59.3)	41(37.7–44.2), [43](39.6–46.3)	32(29.4–34.5), [35](32.2–37.7)
1984–86	90(86.4–93.5), [90](86.4–93.5)	77(72.4–81.5), [74](69.6–78.3)	53(48.8–57.1), [51](47.0–54.9)	38(35.7–40.2), [39](36.7–41.2)	30(28.2–31.7), [31](29.1–32.8)
1987–89	91(87.4–94.5), [91](87.4–94.5)	80(75.2–84.7), [77](72.4–81.5)	54(50.8–57.1), [53](49.8–56.1)	41(38.5–43.4), [40](37.6–42.3)	31(29.1–32.8), [32](30.1–33.8)
1990–92	91(87.4–94.5), [90](86.4–93.5)	77(73.9–80.0), [77](73.9–80.0)	54(50.8–57.1), [57](53.6–60.3)	43(40.4–45.5), [45](42.3–47.6)	37(34.8–39.1), [37](34.8–39.1)
1993–95	91(89.2–92.7), [92](90.1–93.8)	79(75.9–82.0), [79](75.9–82.0)	58(55.7–60.2), [56](53.8–58.1)	44(41.4–46.5), [42](39.5–44.4)	36(34.5–37.4), [34](32.6–35.3)

**Table 6:** Summary of key findings in research for inflammatory breast cancer.

Author	Ref.	Institute	Year	Patient No.	Main results
Palangie	[21]	Institut Curie, France	1977–1987	223	Better DFS – rapid and complete remission with induction treatment
Chang	[22]	MD Anderson, US	1974–1993	177	Shorter OS – for postmenopausal IBC women with larger body size
Liauw	[3]	U. Florida, US	1982–2001	61	Improved CSS – tumor size <4 cm, upfront surgery, local disease control
Perez	[23]	Mallinckrodt, US	1958–1989	179	Improved DFS, CSS, LR – addition of mastectomy
Panades	[24]	British Columbia, Canada	1980–2000	485	Improved LC – mastectomy in addition to chemotherapy and radiotherapy
					Improved CSS – modern chemotherapy regimens
Fleming	[25]	MD Anderson, US	1974–1993	178	Improved LC – mastectomy in addition to radiotherapy 16.3% vs 35.7%, P = 0.015
Buchholz	[26]	MD Anderson, US	1989–1997	12	Chemotherapy with radiation improved OS
Liao	[27]	MD Anderson, US	1977–1993	115	Improved LC, DFS, OS – twice-daily postmastectomy radiation to 66 Gy
Bertucci	[28]	Institut Paoli-Calmettes, France	1976–2000	74	High-dose chemotherapy: 5y DFS 28%, OS 50% Conventional dose chemotherapy: 5y DFS 15%, OS 18%
Ueno	[6]	MD Anderson, US	1973–1993	178	Improved DFS – combined modality: 15y DFS 28%, single-modality: 15y DFS <5%

DFS = disease free survival,

OS = overall survival,

CSS = cause-specific survival,

LC = local recurrence.
